# Tooth Sensitivity Following Hydrogen Peroxide Bleaching With and Without Ozone: A Randomized Controlled Trial: Tooth Sensitivity Following H_2_O_2_ Versus H_2_O_2_/Ozone Bleaching

**DOI:** 10.1155/2024/2695533

**Published:** 2024-10-30

**Authors:** Saeed Awod Bin Hassan

**Affiliations:** Department of Restorative Dental Sciences, College of Dentistry, King Khalid University, Abha, Saudi Arabia

**Keywords:** bleaching, bleaching sensitivity, dentistry, H_2_O_2_, healOzone, hydrogen peroxide, ozone, tooth sensitivity, tooth whitening

## Abstract

**Aims:** The aim of this investigation was to assess bleaching sensitivity following bleaching using either 38% H_2_O_2_ only or 38% H_2_O_2_ followed by ozone application.

**Methods:** In this randomized controlled clinical investigation, 80 participants (40 females and 40 males) were randomly assigned to two groups (*n* = 40 each; 20 females and 20 males). The upper anterior teeth were bleached by 38% H_2_O_2_ for 20 min followed by ozone application for 60 s (healOzone X4, KaVo Dental, Biberach, Germany) in Group 1 (test group). Meanwhile, the bleaching protocol in Group 2 (controls) included the application of just 38% H_2_O_2_ for 20 min. Tooth sensitivity before and after bleaching was reported by the participants using a visual analog scale (VAS) from 0 to 10. Mann–Whitney *U* test, Wilcoxon signed-rank test, and regression analysis were used to analyze the data. Significant statistical outcomes were identified at *p* < 0.05.

**Results:** Bleaching sensitivity was reported following both tested bleaching protocols (*p* < 0.001). However, less bleaching sensitivity was reported when ozone was applied for 60 s after bleaching with 38% H_2_O_2_ (*p* < 0.001). Female participants reported more bleaching sensitivity regardless the applied bleaching protocol (*p* < 0.05).

**Conclusions:** Bleaching protocols with 38% hydrogen peroxide were associated with less bleaching sensitivity when followed by ozone application on the teeth.

## 1. Introduction

Bleaching sensitivity is a common phenomenon following bleaching [1–8]. It was reported to affect up to 70% of patients who received tooth whitening treatments [9, 10]. Bleaching sensitivity is usually temporary and disappears with time [9, 10].

The underlying cause of bleaching sensitivity was attributed to the increased porosity and widening of the openings of dentinal tubules which allow easier access to the pulp [1–3, 8, 11]. Also, it was attributed to the free radicals that result from decomposition of the bleaching materials which induce the release of proinflammatory mediators (e.g., prostaglandins, ATP, and cyclooxygenases) [1–3, 8, 11–15]. In addition, the carriers used in bleaching agents (such as glycerin) would absorb water and potentially lead to dehydration of dentinal tubules leading to tooth sensitivity [9].

Nevertheless, the literature lack evidence-based investigations to explain the bleaching sensitivity phenomenon. Also, the literature demonstrated obvious variations in research methods and findings regarding to its duration and severity [16]. Furthermore, many investigations had high risk of bias based on study design in this regard [16].

Various methods for management of bleaching sensitivity were described in the literature. These include the application of desensitizing agents before or after bleaching (such as fluoride gel, potassium nitrate, and casein phosphor–peptide–amorphous calcium phosphate (CPP-ACP)) [17]. In addition, reduction in the frequency of application and the concentration of bleaching agents was advocated to reduce bleaching sensitivity [4, 8, 13, 18–23]. Furthermore, the use of hybrid light/LED/laser irradiation during bleaching was used to reduce bleaching sensitivity [24–26]. However, some studies showed that using desensitizing agents (e.g., sodium fluoride and potassium nitrate) or activation with light/laser during bleaching with hydrogen peroxide had no effect in reducing bleaching sensitivity [27–31]. In addition, the biomimetic hydroxyapatite [32] and other bioactive compounds, such as bioactive glass and arginine [33], were recently tested and could be used for the management of bleaching sensitivity.

Ozone has been used for dental and oral pain control [34–39] and tooth bleaching [1–3, 40–42]. In dentistry, ozone can be administered in a number of different forms: gel [43], gas [44], or water [45]. Some studies advocated the use of ozone following bleaching with high H_2_O_2_ concentrations to reduce bleaching sensitivity [1–3, 6]. This could be speculated to the anti-inflammatory function of ozone [1–3, 12, 46] and its effects on collagen in dentinal tubules [2, 12, 34–36]. However, some studies showed no effect of ozone if applied alone [47–50] or before bleaching [2].

The literature is short of studies into the potential effects of using ozone on bleaching sensitivity following bleaching with hydrogen peroxide. In addition, many variations between previous studies including study designs and applied methods highlighted the need for further controlled trials in this regard. This inspired carrying out this investigation to explore the effects of ozone on bleaching sensitivity within controlled clinical settings.

The aim of this investigation was to assess bleaching sensitivity following bleaching using either 20 min of 38% H_2_O_2_ only or 20 min of 38% H_2_O_2_ followed by ozone application for 60 s within clinical settings.

The null hypothesis for the current investigation was that bleaching sensitivity following the application of 20 min of 38% H_2_O_2_ only would not be different from bleaching sensitivity after the application of 20 min of 38% H_2_O_2_ followed by ozone application for 60 s.

## 2. Materials and Methods

This randomized controlled investigation was ethically approved by the Institutional Review Board (Deanship of Scientific Research, King Khalid University, Saudi Arabia; reference number: GRP/246/44, date: 21st August 2023) and was not registered in any trial registry. This investigation was conducted in full agreement with the Helsinki Declaration (ninth version, 2013). The manuscript was prepared following the guidelines of the CONSORT statement on reporting randomized trials. All participants were requested to sign a written informed consent before participation. The participants were recruited from the dental clinics of the Department of Restorative Dental Sciences/School of Dentistry at King Khalid University between December 2022 and March 2023.

**Figure 1:**
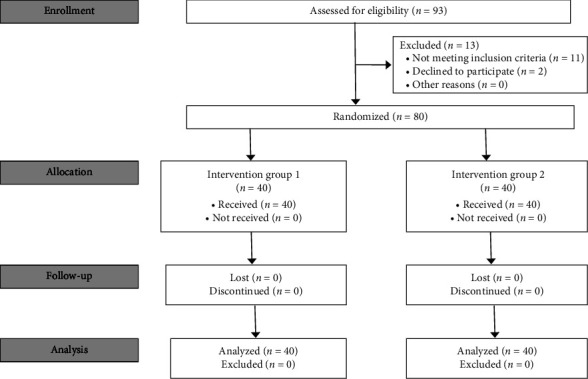
Study flow of participants (Group 1 = teeth bleached with 38% hydrogen peroxide then ozone and Group 2 = teeth bleached with 38% hydrogen peroxide alone).

Overall, 93 participants who agreed to participate in this investigation were assessed (Figure 1). Thirteen participants were excluded because they did not meet the inclusion criteria or declined to participate. In total, 80 participants (40 females and 40 males) were ultimately included in this investigation, and none was lost during the investigation (no drop out) (Figure 1).

To compute the sample size for this investigation, a priori power analysis was performed utilizing the Mann–Whitney test based on 0.8 study power, *α* of 0.05 and effect size of 0.5 (G ∗ Power version 3.1.9.7: Statistical Power Analyses; Heinrich-Heine University, Düsseldorf, Germany). The effect size for this study was determined based on a previous study by Al-Omiri et al. [2]. Bleaching sensitivity was considered the primary outcome measure in this regard. The computed sample size was 68 participants (34 for each group). However, 80 participants were included in the study to compensate for any potential drop out of participants and to warrant having the required sample throughout the investigation. Also, having further participants would potentially improve the confidence in the results and was covered by the available research funds.

For inclusion in this investigation, the participants had to have all their upper six anterior teeth present, vital and sound. Also, they should have received no prior bleaching, orthodontic, restorative, or prosthetic treatment. In addition, their upper anterior teeth should not be affected with caries, periodontal disease, tooth wear, tooth fractures, tooth sensitivity, or endodontic problems. The participants should also be 19–25 years of age to be included.

Participants who had prior bleaching, orthodontic, restorative, or prosthetic treatment were excluded in this investigation. Participants who had one or more of their upper anterior teeth missing, nonvital or affected by caries, periodontal disease, tooth wear, tooth fractures, tooth sensitivity, or endodontic problems were also excluded from this investigation. Also, participants with underlying medical history and nursing/pregnant women were excluded in this investigation.

### 2.1. Randomization and Allocation

After examination and baseline assessment of participants' perception of tooth sensitivity before bleaching, the participants were assigned into two groups (*n* = 40 each). This was based on a gender stratified simple randomization method that employed computer-produced random numbers [1–3]. Each group included 20 males and 20 females.

### 2.2. Concealment

Tooth bleaching protocols were allocated and performed by one investigator. Meanwhile, tooth sensitivity evaluation was reported by another clinician who was blinded to the employed bleaching protocol.

### 2.3. Treatment Protocols

The participants were clinically examined on a dental unit with light (Diamond LED Dental Light; Daray Lighting, Derbyshire, England, United Kingdom) utilizing dental probe and mirror (Hahnenkratt EROGform Explorer and Hahnenkratt Economy Mouth mirror; Königsbach-Stein, Germany) after cleaning and drying the teeth with gauze. A tooth prophylaxis was carried out before the investigation.

Consequently, a visual analog scale (VAS) was employed to evaluate participants' perception of tooth sensitivity before bleaching on a scale from 0 (absence of tooth sensitivity before bleaching) to 10 (most severe sensitivity). After that, the participants were randomly assigned into two groups (*n* = 40 each).

Then, the upper anterior teeth of each participant in each group were bleached after application of light cured gingival dam cover (BMS white BM; BMS Dental, Capannoli, Italy). The bleaching protocol in Group 1 involved application of 38% hydrogen peroxide gel (BMS white 38%; BMS Dental, Capannoli, Italy) on the labial surfaces of the upper anterior teeth for 20 min before washing it away using suction and water spray from a three-in-one syringe. Then, ozone gas was applied to the teeth surfaces for 60 s at 615 cc/minute flow rate and 2,350 ppm concentration. The ozone gas was applied through disposable silicon cups fitted on the handpiece of the ozone supplying appliance (healOzone X4 machine; Curozone, Germany). Meanwhile, the bleaching protocol in Group 2 involved exclusive application of 38% hydrogen peroxide gel on the labial surfaces of the upper anterior teeth for 20 min and then washed away using suction and water spray from a three-in-one syringe.

After bleaching, the participants were released and requested to attend for follow-up after 24 h for assessment of bleaching sensitivity. Then, bleaching sensitivity was reported 24 h after bleaching employing a VAS scale from 0 (*absence of bleaching sensitivity*) to 10 (*most severe bleaching sensitivity*) as described above.

No side effects or harms were detected in any participant during this investigation except bleaching sensitivity which is the main outcome measure of this investigation. However, bleaching sensitivity is a well-known and expected result following bleaching.

Ten duplicate bleaching sensitivity evaluations were performed by the same clinician to appraise the intraexaminer reliability. The Kappa values (*κ* = 0.90) showed proper reliability of the bleaching sensitivity evaluation.

### 2.4. Statistical Analysis

The gathered data from this investigation were managed utilizing the Statistical Package for the Social Sciences (SPSS) computer program (IBM SPSS statistics v19.0; IBM Corp., New York, United States of America). Probability of p < 0.05 at 95% confidence intervals was considered to identify significance during the statistical analysis. The Kolmogorov–Smirnov test demonstrated that age, VAS scores of the levels of tooth sensitivity before bleaching, and VAS scores of the levels of bleaching sensitivity were not normally distributed. Therefore, nonparametric statistical tests were utilized during the analysis, and the median and interquartile range (IQR) war also used to describe age and the levels of sensitivity among the study population before and after bleaching.

Comparisons of the levels of sensitivity between genders and between the test and control groups were performed utilizing the Mann–Whitney test. Within-group comparisons were performed utilizing the Wilcoxon signed-rank test to identify differences between bleaching sensitivity and the levels of sensitivity before bleaching. The strength of the association between the levels of sensitivity and each of the group as well as gender was indicated via the effect size (Eta squared *η*^2^ for Mann–Whitney *U* test and r for Wilcoxon signed-rank test).

A stepwise multiple regression analysis was carried out to distinguish the factors (including bleaching protocol, gender, and age) that would be capable of predicting bleaching sensitivity. The analysis considered the confounding effects of gender and age.

## 3. Results

The data collected from 80 participants (40 females and 40 males) were evaluated and analyzed. Participants' age ranged from 19 to 25 years (mean [±SD] = 21.49 [±1.92] years, Table 1). No significant difference in age was observed between sexes (Mann–Whitney *U* = 26,136.0, *z* statistic = −1.776, and *p*=0.076) nor between test and control groups (Mann–Whitney *U* = 28,656.0, *z* statistic = −0.096, and *p*=0.924).

Table 1 also presents the descriptive statistics for the VAS scores for bleaching sensitivity and tooth sensitivity before bleaching according gender and bleaching protocol among the study population. None of the participants had tooth sensitivity before bleaching (mean VAS score = zero). However, bleaching sensitivity was reported among the participants in both groups following bleaching (Table 1).

The participants reported significant bleaching sensitivity among the total study sample, among the test group (bleaching with H_2_O_2_ then ozone), and among the control group participants (bleaching with H_2_O_2_ alone) (*p* < 0.001, Table 2). Also, a significant increase in sensitivity was reported among females (increased sensitivity in 55.0% of study female participants, Wilcoxon signed-rank test *z* statistic = −10.010, and *p* < 0.001) and males (increased sensitivity in 43.3% of study male participants, Wilcoxon signed-rank test *z* statistic = −8.953, and *p* < 0.001) following bleaching in this study. Further comparisons within each group showed that more sensitivity was reported by each gender after bleaching (*p* < 0.05, Table 2).

Table 3 presents comparisons of bleaching sensitivity between bleaching protocols and between genders among the study population. Considering the bleaching protocol, bleaching with H_2_O_2_ followed by ozone was associated with significantly less bleaching sensitivity in comparison to bleaching with H_2_O_2_ only (Mann–Whitney *U* = 7680.0, *z* statistic = −14.966, and *p* < 0.001, Table 3). Considering participants' gender, female participants reported higher levels of bleaching sensitivity than males among the total study sample, among the test group (bleaching with H_2_O_2_ then ozone), and among the controls (bleaching with H_2_O_2_ alone) (*p* < 0.05, Table 3).

A stepwise multiple regression analysis including bleaching protocol, gender, and age demonstrated that a bleaching protocol with H_2_O_2_ alone (*R*^2^ = 0.361, *B* = 2.558, SE of *B* = 0.163, Beta = 0.574, *t* = 15.684, *p* < 0.001, and 95% confidence intervals for *B* = 2.238–2.879) and being a female (*R*^2^ = 0.361, *B* = 0.792, SE of *B* = 0.163, beta = 0.178, *t* = 4.853, *p* < 0.001, and 95% confidence intervals for *B* = 0.471–1.112) were accompanied with higher odds of having more severe bleaching sensitivity. Therefore, bleaching protocol and gender were considered as adequate predictors for bleaching sensitivity.

A post hoc analysis for the achieved actual power of the study was conducted (G ∗ Power version 3.1.9.7: Statistical Power Analyses; Heinrich-Heine University, Düsseldorf, Germany). It utilized the Mann–Whitney *U* test with an effect size of 0.4676 (Table 3), sample size of 80, and *α* of 0.05 and revealed that the actual achieved study power was 0.82.

## 4. Discussion

The null hypothesis for this investigation was rejected based on the study findings. The results revealed that the exclusive application of 20 min of 38% H_2_O_2_ for tooth bleaching was associated with more bleaching sensitivity than the application of 20 min of 38% H_2_O_2_ followed by ozone application for 60 s.

For this investigation, the healOzone device was used since it was proved to generate, contain, and deliver the required concentrations and flow rates of ozone safely and was used within similar clinical settings before [1–3, 38, 39]. It delivered ozone to the tooth surface through disposable silicon cups that secured complete sealing to prevent ozone gas leakage. The machine was safe to use within clinical settings because it would only work and deliver ozone if the system was sealed perfectly [1–3, 38, 39].

The outcomes of the current investigation revealed that bleaching sensitivity was decreased when ozone was applied after hydrogen peroxide. This agrees with the outcomes of previous studies [1–3]. This could be due to pain control properties of ozone [1–3, 34–42, 51], decreased number and diameter of open dentinal tubules due to collagen degradation via ozone [2, 12, 34–36], and some enamel surface remineralization [52, 53]. Also, this could be due to the anti-inflammatory function of ozone which could reduce prostaglandin manufacturing, reduce the activity of cyclooxygenase, and enhance the activity of antioxidant enzymes such as glutathione peroxidase and superoxide dismutase, and thus counteract the effects of hydrogen peroxide on the tissues [1–3, 46].

The tested bleaching combination in this study was selected to be the protocol of application of hydrogen peroxide followed by ozone application because it was established to be effective by previous studies [1–3]. Furthermore, ozone was found to have no effects on sensitivity reduction if applied before hydrogen peroxide [2]. Besides, ozone alone was not found to be effective on the long term in treatment of dentine hypersensitivity caused by other reasons than bleaching [47–50]. However, exclusive ozone application was associated with less pain on the short term and immediately after application [47–49]. Also, simultaneous application of both ozonated olive oil and calcium sodium phosphosilicate wash was associated with less tooth sensitivity caused by other reasons than bleaching [51].

This variation could be due to various factors. It could be due to variations in designs of studies, applied ozone concentrations, frequency of applications, durations of applications, administration of adjunct materials with ozone, and sequence of administration of bleaching materials [2, 16, 47–51]. Also, it could be due to differences in methods used to evaluate pain (tactile stimulus, desiccation, or participant's perception of pain levels), and whether the pain was evaluated immediate or long term following ozone application [2, 16, 47–51].

The exclusive use of hydrogen peroxide for bleaching is known to be associated with bleaching sensitivity [1–5, 8–11, 16]. This is in agreement with the outcomes of this investigation. This could be due to that hydrogen peroxide breaks down and liberates protons (H^+^) that create minute openings in the enamel surface, and thus making the tooth more prone to sensitivity [52, 53]. In addition, hydrogen peroxide is known to boost the expression of inflammatory mediators such as substance P, prostaglandins, and cyclooxygenases that increase pain stimulation and cause local inflammation of the pulp [12, 54]. Nevertheless, the explanation of the bleaching sensitivity phenomenon still lacks sound evidence–based literature [16]. This is further complicated by differences in research methods and results as well as high risk of bias of many studies [16].

Consequently, application of ozone following hydrogen peroxide during bleaching could potentially reduce the concentrations, durations, and frequencies of hydrogen peroxide applications. This could potentially decrease the possibility of bleaching sensitivity and tissue irritation, reduce the required concentrations for hydrogen peroxide, enhance patients' compliance, and reduce treatment duration and costs [1–3, 7, 52, 53, 55]. In addition, ozone does not cause sensitivity, is associated with less soft tissue irritation, easily controlled by new ozone generating appliances, can be administered with other bleaching materials and products, easily applied, and less costly [1–3]. Also, ozone can be administered in various forms including gas, solution, and gel forms [43–45]. This would offer the required versatility for patient compliance and favorable treatment outcomes.

The outcomes of this study showed that females had more sensitivity after bleaching than males. This is consistent with previous studies that showed females to have more dentine hypersensitivity than males [55–57]. This might be attributed to that females tend to over-report sensitivity to underlying medical conditions [58]. Also, this might be speculated due to that they apply meticulous plaque control which might increase the chance for sensitivity [58, 59]. In addition, they might have smaller size of teeth which have reduced thickness of enamel and dentine, and thus might be more vulnerable to sensitivity [55, 58, 59]. However, some researchers found no significant difference between genders regarding tooth sensitivity although they found some tendency for sensitivity among females [59]. This highlights the need for further research in this regard.

During this investigation, young participants (aged 19–25 years old) were selected for this study to avoid age effects on the dental tissues including the atrophy of pulp tissues and the deposition of dentine [55, 60, 61]. The previous research has shown that young individuals might demonstrate more sensitivity than old ones [55, 60, 61]. Therefore, inclusion of older participants was avoided to avoid any potential bias of the results. However, some researchers found that age was not related to dentine sensitivity [56]. Further research to compare bleaching sensitivity between young and old participants is recommended.

The priori estimated study power for this study was 0.80. After completion of the study, the achieved actual study power was 0.82. This indicates that the study had adequate power. Also, the study was randomized and blinded. Therefore, the confidence in the reported outcomes is considered adequate, and conclusions were achieved based on the obtained study outcomes.

A limitation of this investigation is that it evaluates the short-term and immediate impacts of ozone on bleaching sensitivity after bleaching with hydrogen peroxide. This might not fully reflect the treatment's clinical utility and potentials, and further investigations of the long-term assessment of bleaching sensitivity are advocated. However, the study was conducted on a larger sample than previous studies in this regard [1–3, 62], randomized, blinded, and explored the associations between gender and bleaching sensitivity. Furthermore, bleaching sensitivity is usually temporary and disappears with time, regardless the bleaching protocols or materials [9, 10]. This emphasizes the applicability of using ozone to reduce sensitivity on the short term when sensitivity is usually the most severe and reducing sensitivity is most needed. Therefore, studying the short-term effects of ozone is still useful and valid. Also, one hydrogen peroxide concentration and one ozone application protocol were tested in this study, and testing other hydrogen peroxide concentrations and different ozone application protocols would have provided further insights; and thus, further research is recommended in this regard. In addition, the study was conducted on one population, and comparison to other populations is recommended in the future research. This investigation tested one form of ozone application (gas), and further tests on other forms of ozone application (gel and water) are advocated.

Future clinical studies and research are also recommended to assess effects of using ozone with hydrogen peroxide on bleaching sensitivity among different populations with various age groups, genders, and racial backgrounds. Furthermore, further tests are recommended to investigate different ozone depletion techniques. In addition, future research is advocated to explore patients' compliance, comfort, satisfaction, and perceptions following ozone and hydrogen peroxide bleaching in comparison to exclusive hydrogen peroxide bleaching.

## 5. Conclusions

Within the limitations of this investigation, it was concluded that bleaching protocol that includes the application of ozone following bleaching with 38% hydrogen peroxide would be associated with lower levels of bleaching sensitivity. Also, females would experience higher levels of bleaching sensitivity than males regardless the utilized bleaching protocols in this investigation.

## Figures and Tables

**Table 1:** Descriptive statistics for bleaching sensitivity and age among the study population (*n* = 80 and 40 participants per group).

Variable	Sample	Descriptive statistics							
		Mean	SE	SD	95% CI	Median	IQR	Min	Max
Age	All samples (*n* = 80)	21.49	0.088	1.92	21.32–21.66	21.00	3	19	25
	Males (*n* = 40)	21.63	0.120	1.86	21.39–21.86	21.00	3	19	25
	Females (*n* = 40)	21.35	0.127	1.97	21.10–21.60	21.00	4	19	25

1VAS scores of bleaching sensitivity	All samples (*n* = 80)	1.53	0.102	2.231	1.329–1.729	0.00	2.0	0	9
	All test groups (*n* = 40)	0.25	0.043	0.663	0.166–0.334	0.00	0.0	0	3
	Females in test groups (*n* = 20)	0.30	0.059	0.643	0.184–0.416	0.00	0.0	0	2
	Males in test groups (*n* = 20)	0.20	0.062	0.681	0.077–0.323	0.00	0.0	0	3
	All control groups (*n* = 40)	2.81	0.161	2.500	2.491–3.126	2.00	4.0	0	9
	Females in control groups (*n* = 20)	3.55	0.242	2.647	3.072–4.028	3.00	4.0	0	9
	Males in control groups (*n* = 20)	2.07	0.192	2.105	1.686–2.447	1.00	2.5	0	7

Abbreviations: 95% CI = 95% confidence intervals, control group = teeth bleached with H_2_O_2_ alone, IQR = interquartile range, Max = maximum value, Min = minimum value, SD = standard deviation, SE = standard error of mean, and test group = teeth bleached with H_2_O_2_ then ozone.

^1^All baseline mean VAS scores of sensitivity before bleaching equal zero for each group.

**Table 2:** Paired variations and differences in VAS scores of bleaching sensitivity and tooth sensitivity before bleaching among the study population (*n* = 80 and 40 participants per group).

Wilcoxon signed-rank tests for paired variations of tooth sensitivity before and after bleaching1							
Test statistics	All study samples (*n* = 80)	Test group (H_2_O_2_ then ozone)			Control group (H_2_O_2_ alone)		
		All groups (*n* = 40)	Female (*n* = 20)	Male (*n* = 20)	All groups (*n* = 40)	Female (*n* = 20)	Male (*n* = 20)
*Z*	−13.404	−5.339	−4.417	−3.145	−12.325	−9.048	−8.424
*P*	< 0.001	**< 0.001**	< 0.001	0.002	**0.001**	< 0.001	< 0.001
Effect size	0.1872	0.0594	0.0813	0.0412	0.3165	0.3411	0.2957
% TS	49%	**15.0%**	20%	10.0%	**83.3%**	90.0%	76.7%

Abbreviations: % TS = percentage of participants with increased sensitivity, control group = teeth bleached with H_2_O_2_ alone, *P* = two-tailed probability value, test group = teeth bleached with H_2_O_2_ then ozone, and *Z* = *Z* statistic using Wilcoxon signed-rank test.

^1^All baseline mean VAS scores of sensitivity before bleaching equal zero for each group.

**Table 3:** Differences in bleaching sensitivity between groups and genders among the study sample (*n* = 80).

Comparison of bleaching sensitivity between	Mann–Whitney *U* test			
	MWU	Z	*P*	Effect size
Test group and control group (*n* = 80 (40 per group))	7680.0	−14.966	**< 0.001**	0.4676
Males and females in all study samples (*n* = 80 (40 per gender))	23,976.0	−3.418	**0.001**	0.0244
Males and females within the test group (*n* = 40 (20 per gender))	6516.0	−2.049	0.040	0.0176
Males and females within the control group (*n* = 40 (20 per gender))	4770.0	−4.584	< 0.001	0.0879

Abbreviations: control group = teeth bleached with H_2_O_2_ alone, MWU = Mann–Whitney *U* test statistic, *P* = two-tailed probability value, test group = teeth bleached with H_2_O_2_ then ozone, and *Z* = *Z* statistic.

## Data Availability

The data used to support the findings of this study are available from the corresponding author on reasonable request.
